# Head trauma results in manyfold increased risk of multiple sclerosis in genetically susceptible individuals

**DOI:** 10.1136/jnnp-2023-332643

**Published:** 2024-01-11

**Authors:** Eva Johansson, Lars Alfredsson, Pernilla Strid, Ingrid Kockum, Tomas Olsson, Anna Karin Hedström

**Affiliations:** 1 Department of Clinical Neuroscience, Karolinska Institutet, Stockholm, Sweden; 2 Clinical Neuroscience, Karolinska Institutet, Stockholm, Sweden

**Keywords:** MULTIPLE SCLEROSIS, HEAD INJURY, GENETICS, TRAUMATIC BRAIN INJURY, EPIDEMIOLOGY

## Abstract

**Background:**

Large register-based studies have reported an association between head trauma and increased risk of multiple sclerosis (MS). We aimed to investigate possible interactions between head trauma and MS-associated HLA genes in relation to MS risk.

**Methods:**

We used a Swedish population-based case-control study (2807 incident cases, 5950 matched controls with HLA genotypes available for 2057 cases, 2887 controls). Subjects with and without a history of self-reported head trauma were compared regarding MS risk, by calculating ORs with 95% CIs using logistic regression models. Additive interaction between head trauma, HLA-DRB1*1501 and absence of HLA-A*0201, was assessed by calculating the attributable proportion (AP) due to interaction.

**Results:**

A history of head trauma was associated with a 30% increased risk of subsequently developing MS (OR 1.34, 95% CI 1.17 to 1.53), with a trend showing increased risk of MS with increasing number of head impacts (p=0.03). We observed synergistic effects between recent head trauma and HLA-DRB1*15:01 as well as absence of HLA*02:01 in relation to MS risk (each AP 0.40, 95% CI 0.1 to 0.7). Recent head trauma in individuals with both genetic risk factors rendered an 18-fold increased risk of MS, compared with those with neither the genetic risk factors nor a history of head trauma (OR 17.7, 95% CI 7.13 to 44.1).

**Conclusions:**

Our findings align with previous observations of a dose-dependent association between head trauma and increased risk of MS and add a novel aspect of this association by revealing synergistic effects between recent head trauma and MS-associated HLA genes.

**WHAT IS ALREADY KNOWN ON THIS TOPIC**

- Prior large register-based studies have observed an association between head trauma and increased risk of multiple sclerosis (MS).

**WHAT THIS STUDY ADDS**

- Our findings indicate that individuals carrying the main MS-associated HLA risk allele DRB1*15:01 or lacking the protective allele HLA-A*02:01 are more susceptible to the effects of head trauma.

**HOW THIS STUDY MIGHT AFFECT RESEARCH, PRACTICE OR POLICY**

- Our study could prompt further research to uncover the underlying mechanisms linking head trauma, genetic factors and MS development.
- Our findings may also contribute to policy discussions regarding preventive measures and risk assessment strategies.

## Introduction

The risk of multiple sclerosis (MS) reflects a complex interplay between environmental and genetic risk factors. Genetic susceptibility to the disease is mainly located within the human leucocyte antigen (HLA) region. The DRB1*15:01 allele, encoding an HLA class II molecule whose principal function is to present antigens to CD4+T cells, is associated with an approximately threefold increased risk of MS. The most robust genetic protective association in MS is linked to the A*02:01 allele, which encodes an HLA class I molecule responsible for presenting antigens to CD8+T cells.^1 2^Several studies have demonstrated the presence of interactions between these genetic risk factors and several environmental exposures in MS development, such as lung-irritating agents^3 4^ and Epstein-Barr virus (EBV) infection.^5^ The interactions with HLA genes suggest that the mechanisms of these environmental factors relate to the adaptive immune system.^6^ Recent large register-based studies have reported an increased risk of MS following traumatic brain injury,^7 8^ particularly if repeated and requiring longer hospital care.^8^ Traumatic brain injury, following a physical impact to the head, may result in long-lasting dysfunction of the blood–brain barrier and neuroinflammation.^9–11^ Brain-derived antigens entering the peripheral circulation could potentially trigger an adaptive immune response targeting the central nervous system (CNS).^12^ Boxing leads to elevated levels of biochemical markers for neuronal injury in cerebrospinal fluid (CSF).^13^ Experimental spinal cord injury can elicit a CNS autoreactive immune response with CNS inflammation.^14^ Experimental nerve trauma activates myelin-specific T cells with ensuing CNS inflammation, dependent on the genetic set up which includes rodent major histocompatibility antigens of the rat strain.^15^ Finally, non-specific CNS injury in the form of stroke results in increased numbers of myelin reactive T cells in the blood.^16^ Considering the basic functions of MS-associated HLA alleles and acknowledging that trauma to the CNS can expose the immune system to nervous tissue antigens, we hypothesised that an increased risk of MS following head trauma could be related to the individual HLA allele composition. Therefore, in this study, we investigated the association between self-reported head trauma and MS risk and the potential interaction of head trauma with MS-associated HLA risk alleles regarding the risk of developing the disease.

## Methods

### Design and study population

We used a Swedish population-based case-control study on environmental and genetic risk factors for MS (Epidemiological Investigation of MS). The study group comprised the Swedish population aged 16–70 years. Incident cases of MS were recruited via hospital-based and privately run neurology units throughout the country. Cases were diagnosed by local neurologists using the McDonald criteria.^17 18^ For each case, two controls were randomly selected from the national population register in close temporal alignment with the case’s inclusion, matched by age in 5-year age strata, sex and residential area. The study period was April 2005 to April 2015. A more detailed description of study design and methods is given elsewhere.^19^

Information regarding demographic factors, environmental exposures and lifestyle habits was collected using a standardised questionnaire. Questionnaires were obtained from 2807 cases and 5950 controls (response rate 93% and 73%, respectively). Questions that were not completely answered, were complemented by phone or mail. All participants who filled out the questionnaire were asked to provide blood samples. Blood samples were available for 2057 cases and 2887 controls (73% and 49%, respectively, of those who filled out the questionnaire).

### Definition of exposure

Participants were asked if they had ever badly hit their head or had suffered a severe blow to their head. If they had, they were asked whether the head trauma was severe enough to cause loss of consciousness or loss of memory. They were also asked to provide the year and the number of times they had suffered a head trauma during the last 5 years. Head trauma was only considered before the year of disease onset among cases (index year) and the corresponding index year among matched controls. Participants were categorised based on whether they had ever experienced a head trauma. Those who had were further categorised into those who had suffered a head trauma within 5 years prior to index and before this period, respectively.

### Genotyping

*HLA-DRB1* and *HLA-A* alleles were determined at four-digit resolution. Genotyping was performed on the MS replication chip,^20^ and alleles for HLA-*DRB1, HLA-A, HLA-B, HLA-DQA1* and *HLA-DQB1* were imputed with HLA*IMP:02.^21^ Participants were categorised based on the presence or absence of HLA-DRB1*15:01, DRB1*03:01, DRB1*08:01, DRB1*13:03, HLA-A*02:01, B*44:02, B*38:01, B*44:02, DQA1*01:01, DQB1*03:02 and DQB1*03:01, respectively. Other measures a participant who was born in any of the Nordic countries, and whose parents had not immigrated from outside the Nordic countries, was classified as Nordic. Ancestry was dichotomised into Nordic and non-Nordic origin. Based on current and previous smoking habits, smoking was categorised into current, past or never smoking at the index. Adolescent body mass index (BMI) was calculated by dividing self-reported weight in kilograms at age 20 years by self-reported height in metres squared and was categorised into underweight, normal weight, overweight and obesity, according to the cutoffs used by the WHO. We constructed a discrete variable for sun exposure, based on three questions regarding exposure to ultraviolet radiation where each answer alternative was given a number ranging from 1 (the lowest exposure) to 4 (the highest exposure). The numbers were added together into a sun exposure index and subjects were dichotomised into low or high sun exposure based on the median value among controls.^22^ Alcohol consumption was categorised into subgroups based on the amount of alcohol intake per week: low consumption (<50 g/week for women and<100 g/week for men), moderate consumption (50–112 g/week for women and 100–168 g/week for men) and high consumption (>112 g/week for women and>168 g/week for men). We used the same cutoffs as those used by Statistics Sweden, a government agency that produces official statistics.^23^ Among the study participants, 15 cases and 52 controls had missing values for one or more potential confounding variables. To address this, an approach was employed by categorising these missing values under ‘unknown value’ for each relevant confounding variable.

### Statistical analysis

Subjects who reported they had suffered a head trauma were compared with those who had not regarding the risk of MS, by calculating ORs with 95% CIs using logistic regression models.^24^ Potential interactions on the additive scale, defined by departure from additivity of effects, between a history of head trauma, presence of DRB1*15:01, and absence of A*02:01 were assessed by calculating the attributable proportion (AP) due to interaction with 95% CI. The AP between two interacting risk factors reflects the joint effect beyond the sum of the independent effects.

Considering the potential for changes in clinical assessments and practices over the recruitment period, we conducted a sensitivity analysis focusing on the subgroup of participants enrolled during the peak 3-year period (2006–2009). We also performed the analyses among those who were younger than 20 years at disease onset and those who were older, respectively.

All analyses were adjusted for age, sex, residential area, ancestry, smoking, physical activity and adolescent BMI, and for the following HLA alleles which have been independently associated with risk of MS; DRB1*03:01, DRB1*13:03, DRB1*08:01, B*44:02, B38:01, B44:02, DQA1*01:01, DQB1*03:02 and DQBI*03:01 ^1^. We also made adjustment for sun exposure habits and alcohol consumption, but these variables were not retained in the final models since they had no influence on the results. All analyses were conducted using SAS V.9.4.

## Results

Our multivariable analyses on head trauma and MS risk included 2807 cases and 5950 controls, and the interaction analyses comprised 2057 cases and 2887 controls. The mean age at onset was 34.6 years and the median duration from disease onset to the diagnosis was 1.0 year. Characteristics of cases and controls in the overall sample as well as limited to those with HLA data available, by a history of head trauma, are presented in table 1.

**Table 1:** Baseline characteristics in overall sample as well as limited to those with HLA data available, by history of head trauma

Overall sample						
	All		Ever head trauma		Never head trauma	
Case–control status	Cases	Controls	Cases	Controls	Cases	Controls
N	2807	5950	888	1559	1919	4391
Age at index (SD)	34.6 (10.6)	34.6 (10.8)	33.8 (10.8)	33.2 (10.5)	35.0 (10.5)	35.2 (10.8)
Female, n (%)	2016 (72)	4249 (71)	576 (65)	976 (63)	1440 (75)	3273 (75)
Nordic origin, n (%)	2272 (81)	4631 (78)	687 (77)	1204 (77)	1585 (83)	3427 (78)
Ever smoking, n (%)	1501 (53)	2580 (43)	506 (57)	726 (47)	995 (52)	1854 (42)
Pack-years, n (SD)	8.0 (9.3)	7.1 (8.6)	8.0 (9.5)	6.8 (9.0)	8.0 (9.2)	7.2 (8.4)
Physical activity (SD)	2.6 (0.9)	2.7 (1.0)	2.7 (1.0)	2.8 (1.0)	2.6 (0.9)	2.6 (0.9)
Sun exposure index (SD)	6.2 (1.8)	6.5 (1.9)	6.2 (1.8)	6.6 (2.0)	6.2 (1.8)	6.5 (1.9)
BMI at age 20 years, kg/m2 (SD)	22.6 (3.9)	21.9 (3.4)	22.8 (4.2)	22.2 (3.5)	22.5 (3.7)	21.9 (3.4)
**Participants with HLA data available**						
	**All**		**Ever head trauma**		**Never head trauma**	
Case–control status	Cases	Controls	Cases	Controls	Cases	Controls
N	2057	2887	651	733	1406	2154
Age at index (SD)	34.4 (10.6)	35.4 (10.8)	33.6 (10.9)	33.8 (10.6)	34.8 (10.5)	35.9 (10.8)
Female, n (%)	1492 (73)	2168 (75)	435 (67)	495 (68)	1057 (75)	2154 (78)
Nordic origin, n (%)	1678 (82)	2290 (79)	504 (77)	573 (78)	1174 (84)	1717 (80)
Ever smoking, n (%)	1092 (53)	1285 (45)	383 (59)	345 (47)	709 (50)	940 (44)
Pack-years, n (SD)	7.5 (7.6)	6.9 (7.2)	7.4 (7.4)	6.5 (7.1)	8.2 (9.1)	7.1 (7.2)
Physical activity (SD)	2.6 (0.9)	2.7 (1.0)	2.7 (1.0)	2.8 (1.0)	2.6 (0.9)	2.6 (0.9)
Sun exposure index (SD)	6.2 (1.9)	6.6 (2.0)	6.2 (1.9)	6.7 (2.1)	6.2 (1.9)	6.6 (2.0)
BMI at age 20 years, kg/m^2^ (SD)	22.6 (3.9)	21.8 (3.1)	22.7 (4.3)	22.0 (3.3)	22.5 (3.8)	21.7 (3.1)

BMI, body mass index.

Ever having suffered a head trauma was associated with a 30% increased risk of subsequently developing MS (OR 1.34, 95% CI 1.17 to 1.53). Compared with those who had never suffered a head trauma, a reported history of head trauma within 5 years prior to index was associated with a 60% increased risk of MS (OR 1.58, 95% CI 1.26 to 1.98), whereas head trauma before this period rendered an OR of 1.29 (95% CI 1.16 to 1.42) (table 2). Among those who had suffered a head trauma, loss of consciousness or memory loss did not significantly increase the risk further (p=0.17). However, there was a significant trend showing increased risk of MS with increasing number of head impacts (p=0.03). The results remained similar when we performed the analysis limited to those with HLA data (table 2).

**Table 2:** OR with 95% CI of MS among those who reported they have suffered a head trauma, compared with those who have not

Total			
Head trauma	Cases/controls	OR (95% CI)^*^	OR (95% CI)^†^
Never	1919/4391	1.0 (reference)	1.0 (reference)
More than 5 years before index	754/1362	1.28 (1.15 to 1.42)	1.29 (1.16 to 1.42)
Within 5 years before index	134/197	1.58 (1.26 to 1.98)	1.58 (1.26 to 1.98)
HLA data available			
Head trauma	Cases/controls	OR (95% CI)^*^	OR (95% CI)^†^
Never	1406/2154	1.0 (reference)	1.0 (reference)
More than 5 years before index	560/644	1.31 (1.14 to 1.49)	1.32 (1.16 to 1.51)
Within 5 years before index	91/89	1.54 (1.13 to 2.05)	1.56 (1.15 to 2.10)

*Adjusted for age, sex and residential area.

†Adjusted for age, sex, residential area, ancestry, smoking, physical activity and body mass index.

MS, multiple sclerosis.

Regarding the risk of MS, a synergistic effect was observed between DRB1*15:01 and head trauma within 5 years prior to onset (AP 0.40, 95% CI 0.08 to 0.73) as well as between A*02:01 and head trauma within 5 years before index (AP 0.40, 95% CI 0.07 to 0.72) (tables 3–4).

**Table 3:** OR with 95% CI of MS among subjects categorised by HLA-DRB1*15:01 status and a reported history of head trauma

Ever head trauma versus never head trauma					
HLA-DRB1*15:01	Head trauma	Cases/controls	OR (95% CI)^*^	OR (95% CI)^†^	AP (95% CI)
–	–	643/1560	1.0 (references)	1.0 (references)	
–	+	292/526	1.33 (1.12 to 1.58)	1.33 (1.11 to 1.58)	
+	–	763/594	3.13 (2.72 to 3.61)	3.72 (3.13 to 4.43)	
+	+	359/207	4.15 (3.41 to 5.04)	4.93 (3.96 to 6.15)	0.18 (0.01 to 0.35)
**Head trauma before 5 years prior to index versus never head trauma**					
HLA-DRB1*15:01	Head trauma	Cases/controls	OR (95% CI)^*^	OR (95% CI)^†^	AP (95% CI)
–	–	643/1560	1.0 (references)	1.0 (references)	
–	+	251/459	1.31 (1.09 to 1.57)	1.31 (1.09 to 1.57)	
+	–	763/594	3.13 (2.72 to 3.61)	3.72 (3.13 to 4.43)	
+	+	309/185	3.98 (3.24 to 4.88)	4.68 (3.72 to 5.89)	0.14 (−0.05 to 0.33)
**Head trauma within 5 years prior to index versus never head trauma**					
HLA-DRB1*15:01	Head trauma	Cases/controls	OR (95% CI)^*^	OR (95% CI)^†^	AP (95% CI)
–	–	643/1560	1.0 (reference)	1.0 (reference)	
–	+	41/67	1.46 (0.98 to 2.18)	1.45 (0.97 to 2.19)	
+	–	763/594	3.12 (2.71 to 3.60)	3.60 (3.00 to 4.31)	
+	+	50/22	5.48 (3.29 to 9.12)	6.77 (3.97 to 11.6)	0.40 (0.08 to 0.73)

*Adjusted for age, sex and residential area.

†Adjusted for age, sex, residential area, ancestry, smoking, physical activity, body mass index, HLA-DRB1*03:01, DRB1*13:03, DRB1*08:01, A*02:01, B*44:02, B*38:01, B*55:01, DQA1*01:01, DQB1*03:02 and DQB1*03:01.

AP, attributable proportion; MS, multiple sclerosis.

**Table 4:** OR with 95% CI of MS among subjects categorised by HLA-A*02:01 status and a reported history of head trauma

Ever head trauma versus never head trauma					
HLA-A*02:01	Head trauma	Cases/controls	OR (95% CI)^*^	OR (95% CI)^†^	AP (95% CI)
+	–	599/1158	1.0 (references)	1.0 (references)	
+	+	253/404	1.20 (1.00 to 1.45)	1.20 (0.99 to 1.46)	
–	–	807/996	1.58 (1.38 to 1.81)	1.65 (1.43 to 1.91)	
–	+	398/329	2.34 (1.96 to 2.80)	2.39 (1.98 to 2.88)	0.22 (0.06 to 0.39)
**Head trauma before 5 years prior to index versus never head trauma**					
HLA-A*02:01	Head trauma	Cases/controls	OR (95% CI)^*^	OR (95% CI)^†^	AP (95% CI)
+	–	599/1158	1.0 (references)	1.0 (references)	
+	+	218/354	1.18 (0.97 to 1.44)	1.18 (0.96 to 1.46)	
–	–	807/996	1.58 (1.38 to 1.81)	1.66 (1.43 to 1.92)	
–	+	342/290	2.28 (1.89 to 2.75)	2.29 (1.88 to 2.79)	0.22 (0.02 to 0.38)
**Head trauma within 5 years prior to index versus never head trauma**					
HLA-A*02:01	Head trauma	Cases/controls	OR (95% CI)^*^	OR (95% CI)^†^	AP (95% CI)
+	–	599/1158	1.0 (references)	1.0 (references)	
+	+	35/50	1.35 (0.86 to 2.10)	1.29 (0.81 to 2.06)	
–	–	807/996	1.58 (1.38 to 1.82)	1.66 (1.43 to 1.92)	
–	+	56/39	2.81 (1.84 to 4.43)	3.23 (2.07 to 5.03)	0.40 (0.07 to 0.72)

*Adjusted for age, sex and residential area.

†Adjusted for age, sex, residential area, ancestry, smoking, physical activity, body mass index, HLA-DRB1*15:01, DRB1*03:01, DRB1*13:03, DRB1*08:01, B*44:02, B*38:01, B*55:01, DQA1*01:01, DQB1*03:02 and DQB1*03:01.

AP, attributable proportion; MS, multiple sclerosis.

Compared with those without the genetic risk factors who had never suffered a head trauma, the risk of MS was increased 18-fold among those with both genetic risk factors who had suffered a head trauma within 5 years before index (OR 17.7, 95% CI 7.13 to 44.1) (table 5, online supplemental figure 1). We also observed indications of a less pronounced interaction between the genetic risk factors and a history of head trauma before 5 years prior to index (tables 3–5).

10.1136/jnnp-2023-332643.supp1 Supplementary data

**Table 5:** OR with 95% CI of MS among subjects categorised by HLA-DRB1*15:01 status, HLA-A*02:01 status and a history of head trauma

Ever head trauma versus never head trauma					
DRB1*15:01	A*02:01	Head trauma	Cases/controls	OR (95% CI)^*^	OR (95% CI)^†^
–	+	–	262/815	1.0 (references)	1.0 (references)
–	+	+	105/285	1.13 (0.85 to 1.48)	1.16 (0.89 to 1.52)
–	–	–	381/745	1.60 (1.33 to 1.93)	1.57 (1.30 to 1.90)
–	–	+	187/241	2.36 (1.84 to 3.03)	2.34 (1.84 to 2.98)
+	+	–	337/343	3.07 (2.50 to 3.77)	3.51 (2.80 to 4.41)
+	+	+	148/119	3.75 (2.88 to 5.04)	4.51 (3.35 to 6.06)
+	–	–	426/251	5.30 (4.30 to 6.54)	6.18 (4.90 to 7.79)
+	–	+	211/88	6.81 (5.07 to 9.16)	8.69 (6.40 to 11.8)
**Head trauma before 5 years prior to index versus never head trauma**					
DRB1*15:01	A*02:01	Head trauma	Cases/controls	OR (95% CI)^*^	OR (95% CI)^†^
–	+	–	262/815	1.0 (references)	1.0 (references)
–	+	+	92/251	1.13 (0.85 to 1.48)	1.16 (0.88 to 1.54)
–	–	–	381/745	1.60 (1.33 to 1.93)	1.57 (1.30 to 1.91)
–	–	+	159/208	2.36 (1.84 to 3.03)	2.30 (1.78 to 2.97)
+	+	–	337/343	3.07 (2.50 to 3.77)	3.53 (2.81 to 4.42)
+	+	+	126/103	3.75 (2.79 to 5.04)	4.38 (3.20 to 6.00)
+	–	–	426/251	5.30 (4.30 to 6.54)	6.20 (4.91 to 7.83)
+	–	+	183/82	6.81 (5.07 to 9.16)	8.03 (5.85 to 10.1)
**Head trauma within 5 years prior to index versus never head trauma**					
DRB1*15:01	A*02:01	Head trauma	Cases/controls	OR (95% CI)^*^	OR (95% CI)^†^
–	+	–	262/815	1.0 (references)	1.0 (references)
–	+	+	13/34	1.17 (0.61 to 2.24)	1.16 (0.60 to 2.26)
–	–	–	381/745	1.60 (1.33 to 1.93)	1.58 (1.30 to 1.90)
–	–	+	28/33	2.62 (1.55 to 4.43)	2.70 (1.57 to 4.62)
+	+	–	337/343	3.06 (2.49 to 3.76)	3.41 (2.70 to 4.29)
+	+	+	22/16	4.29 (2.22 to 8.30)	5.12 (2.60 to 10.1)
+	–	–	426/251	5.29 (4.29 to 6.53)	6.01 (4.73 to 7.62)
+	–	+	28/6	14.3 (5.84 to 34.9)	17.7 (7.13 to 44.1)

*Adjusted for age, sex and residential area.

†Adjusted for age, sex, residential area, ancestry, smoking, physical activity, body mass index, HLA-DRB1*03:01, DRB1*13:03, DRB1*08:01, B*44:02, B*38:01, B*55:01, DQA1*01:01, DQB1*03:02 and DQB1*03:01.

When we performed the analysis based on those recruited during the period 2006–2009, the overall association between ever having had a head trauma and risk of MS was similar to that from the main analysis (OR 1.34, 95% CI 1.12 to 1.61), as was the interaction between recent head trauma and HLA-DRB1*15:01 (AP 0.44, 95% CI 0.14 to 0.94), and between head trauma and absence of HLA-A*02:01 (AP 0.37, 95% CI 0.01 to 0.83) with regard to MS risk.

The OR of MS associated with ever head trauma was 1.39 (95% CI 1.00 to 1.97) among those who were younger than 20 years at disease onset, and 1.31 (95% CI 1.18 to 1.46) among those who were older. The interaction between head trauma and HLA-DRB1*15:01 was significant in both groups (AP 0.67, 95% CI 0.1 to 1.27, among those younger than 20 years, and AP 0.36, 95% CI 0.01 to 0.72, among those who were older). We were not able to assess the interaction between head trauma and HLA-A*02:01 in different age groups, since there were too few HLA-A*02:01 positive controls in the younger group who had suffered a head trauma.

## Discussion

We observed an increased risk of developing MS following head trauma, particularly if the head trauma occurred within a 5-year time frame before disease onset. We also observed synergistic effects between head trauma and the main MS risk HLA alleles regarding the risk of developing the disease.

Systematic reviews and meta-analyses have reported a significant association between head trauma and MS based on high-quality case-control studies, whereas the pooled results from cohort studies have not.^25 26^ However, recent large register studies have observed a dose-dependent relationship between head trauma in adolescence and increased risk of MS.^8 9^ One of these studies, which used prospectively recorded data, revealed no association between non-CNS trauma and risk of MS.^9^ This argues against an option that individuals in the prodromal phase of MS, in which serum neurofilament light chain levels are elevated,^27^ are more prone to a variety of accidents. Consequently, reverse causation is unlikely to be the sole explanation for the observed link between head trauma and MS risk.

Following head trauma, non-specific injury can result in the release of CNS components into the CSF, which can potentially enter the systemic compartment. In response, adaptive immune responses against CNS antigens may occur either in the CNS draining lymph nodes or within the CNS itself.^12^ Furthermore, traumatic brain injury may result in long-lasting blood–brain barrier dysfunction, enhancing the entry of immune cells into the CNS and neuroinflammation.^9–11^ Even minor repetitive CNS trauma has been associated with neuroinflammation and delayed neurological outcomes.^28 29^

Our findings suggest that individuals with a genetic predisposition to developing MS are more susceptible to the effects of head trauma. Although further research is required to clarify the underlying mechanisms, it is possible that head trauma act as a triggering event that activates adaptive immune responses in susceptible individuals. In experimental autoimmune encephalomyelitis, MHC allelic differences in the capacity of the class II molecule to bind CNS autoantigenic peptides is decisive for any ensuing neuroinflammation. If this phenomenon also applies to humans, carriers of the HLA-DRB1*15:01 allele may have a greater tendency to recognise CNS antigens that are released or exposed due to the trauma.^30^ This would align to the observations in studies of experimental nerve trauma, suggesting that certain MHC molecules may be more prone to recognise critical CNS encephalitogenic antigens.^31^ There is also some evidence to suggest that HLA-A*02:01 plays a role in promoting immune tolerance to CNS antigens^32^ although recent data suggests that the protective association of A*02:01 with MS predominantly relate to effects of the type 1 interferon system.^33^

Strong epidemiological evidence supports the role of EBV as a prerequisite for the onset of MS.^34^ However, the high prevalence of EBV within the general population indicates that EBV alone is insufficient to cause the disease. In our current study, we were unable to determine the temporal relationship between EBV infection and head trauma. Nevertheless, head trauma conferred an increased risk of MS in individuals both below the age of 20 and those older. Our findings align with the concept of the sufficient cause model proposed by Rothman.^35^ According to this model, a disease occurs when multiple risk factors act together and reach a threshold level of causality, which then trigger the disease process.

Our study was conducted as a population-based case-control study and information on exposures was collected retrospectively, introducing the potential presence of recall bias. To minimise the potential influence of recall bias, we focused on including cases who had received their diagnosis within the past year. Given the inconsistent findings from previous studies on the association between head trauma and MS risk, it is unlikely that the quality of reported information on head trauma would differ significantly between cases and controls due to different perceptions regarding the potential effects of head trauma. Also confounding factors may be subjected to some degree of recall bias/misclassification bias, for example, self-reported adolescent BMI. However, strong correlations have been observed between self-reported and objectively measured historical weight^36^ and there is no compelling rationale to expect that this would differ in other Western populations. We cannot totally rule out some degree of bias due to misclassification, but it is unlikely that this would have a profound influence on our estimated association between head trauma and MS risk, including the observed presence of gene–environment interaction. It is highly unlikely that misclassification bias is associated with the studied genetic factors. The same is also relevant for unmeasured confounders.

The possibility of reverse causation arises if individuals in the prodromal phase of MS are more prone to experiencing head trauma. While reverse causation cannot be completely ruled out in our study, there is growing evidence supporting a genuine association between head trauma and risk of MS, including the finding that head trauma, but not non-CNS trauma, is associated with increased risk of MS.^8^ In addition, we consider it unlikely that bias would have a substantial impact on our findings, since such a bias would depend both on HLA genotype and a history of head trauma.

Another potential concern is that the recruitment of cases and controls may have introduced selection bias. However, the Swedish healthcare system offers free medical services to all citizens, and it is highly likely that almost all cases of MS are referred to neurology units. Therefore, the possibility of unidentified MS cases significantly biasing our calculations is considered to be low. Selection bias among controls is also likely to be modest since controls were selected from the general population. Additionally, the reported prevalence of lifestyle habits, such as smoking, was consistent with that of the general population in similar age groups.^19^ Furthermore, there were no significant differences with respect to age, sex or frequency of head trauma between those who provided a blood sample and those who did not, indicating that selection bias did not take place in this step.

In conclusion, our findings align with previous observations of a dose-dependent association between head trauma and increased risk of MS. We also observed synergistic effects between head trauma and MS-associated HLA risk alleles in relation to MS risk. Further research is needed to elucidate the underlying mechanisms and explore preventive strategies.

## Data Availability

Data are available on reasonable request. Anonymised data will be shared by request from any qualified investigator that wants to analyse questions that are related to the published article.
