# Supplementary figures and images for: Head trauma results in manyfold increased risk of multiple sclerosis in genetically susceptible individuals
Source: J Neurol Neurosurg Psychiatry. 2024 Jan 11;95(6):554–60. doi: 10.1136/jnnp-2023-332643 (PMC11103305; doi:10.1136/jnnp-2023-332643)

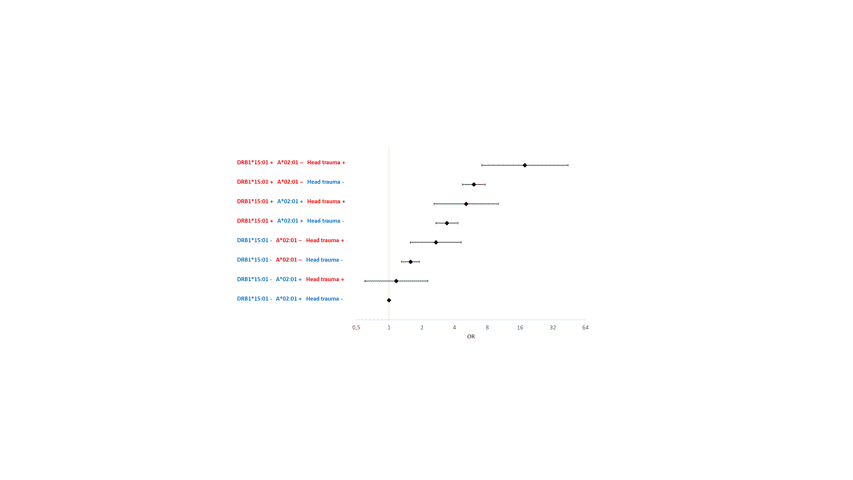

Supplement: Supplementary data [file jnnp-2023-332643supp001.gif]
